# Dysphagia in Rare Diseases and Syndromes: Current Approaches to Management and Therapeutic Innovations—A Systematic Review

**DOI:** 10.3390/healthcare13010052

**Published:** 2024-12-30

**Authors:** Soultana Papadopoulou, Areti Anagnostopouplou, Dimitra V. Katsarou, Kalliopi Megari, Efthymia Efthymiou, Alexandros Argyriadis, Georgios Kougioumtzis, Maria Theodoratou, Maria Sofologi, Agathi Argyriadi, Efterpi Pavlidou, Eugenia I. Toki

**Affiliations:** 1 Department of Speech Therapy, School of Health Sciences, University of Ioannina, 455 00 Ioannina, Greece; efterpipavlidou@uoi.gr (E.P.); toki@uoi.gr (E.I.T.); 2 Department of Preschool Education Sciences and Educational Design, University of the Aegean, 851 32 Rhodes, Greece; a_anagno@yahoo.gr (A.A.); d.katsarou@aegean.gr (D.V.K.); 3 School of Education, University of Nicosia, 2417 Nicosia, Cyprus; 4 City College, University of York, Europe Campus, 546 22 Thessaloniki, Greece; kmegari@psy.auth.gr; 5 College of Interdisciplinary Studies, Zayed University, Dubai 144534, United Arab Emirates; efthymia.efthymiou@zu.ac.ae; 6 Department of Nursing, School of Health Sciences, Frederick University, 3080 Limassol, Cyprus; hsc.arg@frederick.ac.cy; 7 Department of Turkish Studies and Modern Asian Studies, Faculty of Economic and Political Sciences, National and Kapodistrian University of Athens, 105 59 Athens, Greece; georgetype@gmail.com; 8 Department of Psychology, School of Health Sciences, Neapolis University, 8042 Pafos, Cyprus; mttheoria3@gmail.com; 9 School of Social Sciences, Hellenic Open University, 263 35 Patra, Greece; 10 Department of Early Childhood Education, School of Education, University of Ioannina, 451 10 Ioannina, Greece; m.sofologi@uoi.gr; 11 Department of Psychology and Social Sciences, Frederick University, 3080 Limassol, Cyprus; pre.aa@frederick.ac.cy

**Keywords:** dysphagia, rare diseases, syndromes, speech language pathology, speech language therapy, therapeutic, intervention

## Abstract

**Background:** This study presents a comprehensive investigation into the correlation between Rare Diseases and Syndromes (RDS) and the dysphagic disorders manifested during childhood and adulthood in affected patients. Dysphagia is characterized by difficulty or an inability to swallow food of any consistency, as well as saliva or medications, from the oral cavity to the stomach. RDS often present with complex and heterogeneous clinical manifestations, making it challenging to develop standardized diagnostic and therapeutic approaches. Dysphagia can arise from various etiologies, including those related to the central nervous system, inflammatory and neoplastic processes, anatomical or structural disorders, and neuromuscular conditions. These diverse etiologies can result in both structural and functional deficits or neurological impairments that compromise swallowing function. While RDS frequently leads to uncommon conditions, dysphagia remains an underrecognized complication. **Objectives**: The primary objective of this review is to illuminate the latest knowledge concerning the management of dysphagia in both pediatric and adult populations within the context of RDS, with a particular focus on current therapeutic approaches. To achieve this, the study provides a comprehensive analysis of existing strategies for managing dysphagia in RDS, highlighting recent advancements in therapy while identifying critical gaps in clinical knowledge and practice. By synthesizing available evidence, the review aims to deepen understanding of the unique challenges associated with dysphagia in these conditions and explore innovative interventions to enhance patient care and outcomes. **Results:** The integration of innovative therapeutic techniques into the speech-language pathology treatment of dysphagia augments traditional strategies, offering updated knowledge that can be applied to prognosis and therapeutic interventions across various ages and racial groups. This review also provides an overview of symptomatology, assessment techniques, and the specific characteristics of dysphagia associated with various genetic and acquired RDS.

## 1. Introduction

Swallowing is a complex process that involves neuromuscular, sensorimotor, and cognitive functions. As a critical physiological function, swallowing is vital in sustaining human life by performing three essential tasks: airway protection, nutrition, and hydration. Additionally, it significantly contributes to maintaining overall well-being and quality of life [1].

The World Health Organization (WHO) has identified dysphagia—difficulty in swallowing—as a medical impairment associated with increased morbidity, mortality, healthcare costs, and the need for rehabilitation. Dysphagic symptoms arise from disturbances in the oral cavity, pharynx, esophagus, or proximal stomach and are categorized as either oropharyngeal or esophageal dysphagia [2].

On the other hand, neurological dysphagia is caused by neuromuscular abnormalities or lesions in various regions of the central nervous system. Its management is considered a long-term commitment, with complete resolution almost unattainable. Treatment primarily focuses on addressing the underlying neurological damage, whether through surgical or pharmaceutical means, followed by applying the core principle in dysphagia treatment: optimizing preserved functions to compensate for deficits caused by the affected areas [3].

Imaging is the cornerstone of assessing swallowing disorders, often with radiological examinations. Flexible endoscopic evaluation, ultrasound, manometry, electromyography, and scintigraphy are additional diagnostic tools that complement the comprehensive assessment of swallowing disorders by addressing various structural and functional aspects of the swallowing mechanism. The videofluoroscopic study of swallowing (VFSS), also known as a modified barium ingestion study, is a radiographic procedure that provides a direct, dynamic picture of the function of the mouth, pharynx, and upper esophagus. Although speech-language pathologists scientifically investigate and treat the oropharyngeal stage of swallowing, a multidisciplinary team that remedies swallowing issues is necessary to optimize intervention results [3,4].

RDS, defined as conditions affecting fewer than 1 in 2000 individuals in the EU or fewer than 200,000 in the U.S., represent a diverse group of disorders, including autoimmune diseases, genetic syndromes, and certain cancers. Often chronic, progressive, and life-threatening, their etiologies encompass genetic mutations, infections, degenerative mechanisms, and complex biological processes. The rarity of these RDS poses challenges in research, diagnosis, therapy development, and healthcare resources, frequently leading to unmet clinical needs. Studies addressing RDS, particularly those linked to dysphagia, are crucial for advancing understanding and management. The rarity of these conditions often results in them being poorly understood, researched, and recognized, posing challenges in diagnosis and treatment. Addressing RDS typically involves collaboration among researchers, multidisciplinary patient advocacy groups, and policymakers to enhance detection, diagnosis, and treatment options for those affected [2].

The primary objective of the current study is to investigate the clinical symptoms and management strategies of dysphagia across various RDS. Additionally, this study aims to demonstrate that effective dysphagia management can alleviate clinical symptoms and aid in the functional recovery of affected areas, thereby providing relief from the complications associated with RDS. Even though awareness is rising, there are still a lot of unanswered questions about dysphagia in uncommon diseases. A thorough evaluation is necessary because of the wide range of symptoms, the absence of established diagnostic standards, and the scarcity of available treatments. The long-term results of managing dysphagia in these patients are also little documented [4].

Finally, early clinical assessment for feeding disorders is emphasized, alongside the development of significant clinical indicators and their targeted optimal management [5]. Improving patient outcomes requires filling in these knowledge gaps. The efficient management of dysphagia in patients with uncommon disorders can result in better nutritional status, lower medical expenses, and an enhanced quality of life. Additionally, a thorough understanding can help medical practitioners make well-informed decisions regarding patient care.

This systematic review aims to gather and evaluate the information currently available on the frequency, diagnosis, clinical presentation, treatment, and outcomes of dysphagia treatments in patients with RDS. Given the heterogeneity in clinical manifestations and the lack of clinical guidelines, this study aims to provide guidance for understanding the mechanisms of dysphagia and diagnosing and treating dysphagia RDS by demonstrating the effectiveness of the proposed therapeutic measures. In this way, it will shed light on aspects that have not been sufficiently researched, covering bibliographic gaps and helping those involved with RDS scientific disciplines.

## 2. Materials and Methods

### 2.1. Research Questions

This systematic review was designed to explore two primary research questions:

1. Are RDS associated with feeding and swallowing disorders?
2. What modern therapeutic methods effectively treat feeding disorders associated with RDS?

In this systematic review of dysphagia and RDS, the following specific questions are addressed:

1. What are the primary pathophysiological processes that cause dysphagia in RDS?
2. What RDS are most frequently linked to dysphagia, and how do they impact the ability to swallow?
3. What are the typical symptoms, and how does dysphagia present clinically in patients with RDS?
4. When evaluating dysphagia in patients with RDS, which diagnostic methods and instruments work best?
5. Which therapies are available, and how successful are they at treating dysphagia in people with RDS (innovative methods such as neuromuscular electrical stimulation and biofeedback techniques, and personalized interventions tailored to patients with RDS)?
6. In the context of RDS, what are the present obstacles and knowledge gaps regarding dysphagia, and what areas need more investigation?
7. What research priorities should guide future efforts in this area to optimize patient care?

### 2.2. Search Strategy and Inclusion Criteria

This study follows the Assessment of Multiple Systematic Reviews (PRISMA 2020) framework guidelines [6].

A comprehensive literature search was conducted using the Science Direct and PubMed databases. The search strategy implemented on Science Direct included the following query: “deglutition” OR “swallowing disorders” OR “dysphagia” OR “deglutition disorders” OR “swallowing difficulties” OR “rare disorders” OR “rare syndromes” OR “rare diseases” OR “case studies” AND (speech and language therapy OR pathology) AND “dysphagia management”. This query was iteratively refined to capture the most relevant studies.

All retrieved articles underwent a multi-step screening process, starting with removing duplicates and reviewing titles and abstracts for relevance. Full-text articles were then assessed based on the inclusion criteria. Two independent reviewers performed the screening, and discrepancies were resolved through discussion or consultation with a third reviewer.

To be eligible for inclusion, publications must meet the following criteria regarding the type of publication (only original research, review, and case study articles), the language of publication (only English), the publication date (last ten years), and the content (therapeutic approaches for RDS in the context of dysphagia).

Data was extracted from the selected studies, focusing on the patient’s clinical characteristics, type of dysphagia, underlying RDS, and proposed therapeutic interventions. A quality assessment of the studies was conducted, focusing on the risk of bias and overall study quality.

Table 1 summarizes the search strategy, inclusion criteria, and key details of the methods used in this systematic review.

### 2.3. PRISMA Flow Diagram

**Figure 1:**
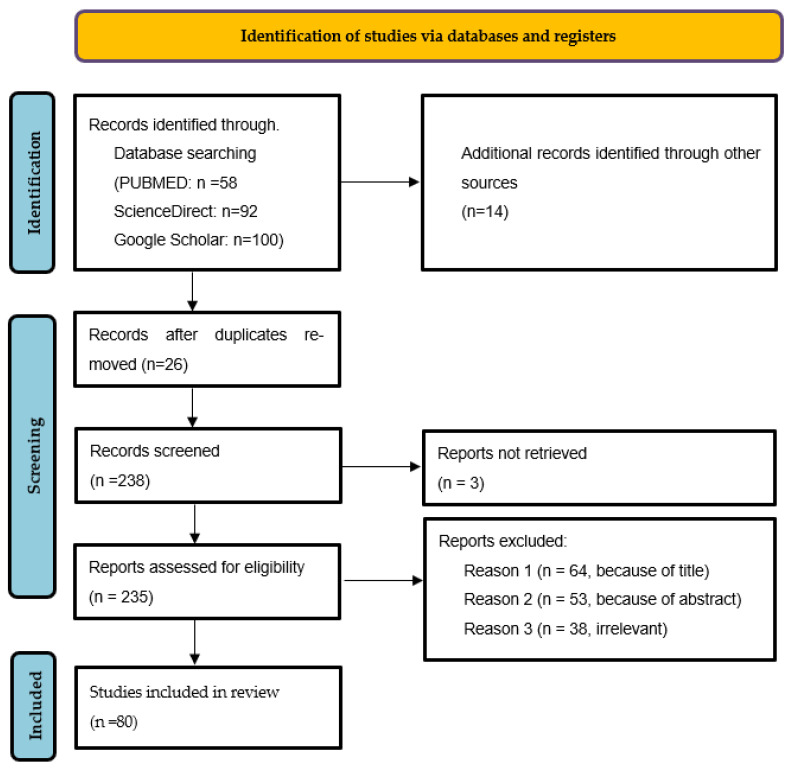
PRISMA 2020 Flow Diagram.

Figure 1 includes the PRISMA flow diagram to provide a clear overview of the study selection process. This diagram details the number of records identified, screened, and included in the review.

### 2.4. Data Extraction

To answer the research questions, extracting relevant information from the 86 publications in the established corpus was crucial. The extraction process focused on several key aspects, including the geographic location, activity sectors, objectives pursued, methodologies used, and obtained outcomes. This ensured that the necessary data was gathered and analyzed to effectively address the research questions.

## 3. Results

### Dysphagia and RDS

Many of the RDS discovered have common problems, such as dysphagia and cognitive abnormalities, that necessitate the diagnosis and treatment of a speech-language pathologist.

*Plummer-Vinson syndrome (PVS)*, also known as Patterson-Brown-Kelly syndrome and sideropenic dysphagia, is classified as a rare disease by the National Institutes of Health’s (NIH) Office of Rare Diseases (ORD). Plummer-Vinson’s disease is becoming less common over time. It is rarely accompanied by episodes of dysphagia, primarily in the geriatric population (10% of the total), with manifestations such as continuous gastresophageal reflux, reduced appetite, remarkable weight loss in a short period (4.5 kg in one year), and difficulty swallowing solid foods. These are frequently connected with esophageal stricture, neurological problems, and anemia. The patient is led to starvation and, in extreme cases, death. An esophagogastroduodenoscopy and barium swallow test are used to diagnose the related strictures. Iron replacement therapy or surgically opening the stricture (dilating the esophageal meshwork) can alleviate symptoms. However, iron supplementation may not always help with [7].

Oropharyngeal dysphagia is likely to be a key feature of *Niemann-Pick disease type C (NP-C)*, a rare genetic disorder that affects lipid metabolism, leading to the accumulation of lipids in various tissues, including the brain (NP-C), and increases the risk of aspiration leading to bronchopneumonia [8]—a significant cause of death in the syndrome in general [9]. The most likely clinical indicators are a delayed swallowing reflex and language impairment. Therapeutically, the Miglustat technique, which is the only one approved to date for the treatment of the neurological symptoms of NP-C in the pediatric and non-pediatric populations, stabilizes swallowing disorders, lowering the risk of aspiration over time (up to 66 months) [8].

The main symptom of *Stüve-Wiedemann syndrome* is an oropharyngeal dysfunction in the swallowing process, which raises the risk of aspiration and subsequent pneumonia. The following symptoms are caused by an inability to control the C1–C5 cervical roots, resulting in pharyngo-esophageal dyskinesia [10].

*Moebius syndrome* is characterized by cranial nerve IX and X paralysis as well as laryngotracheal dysfunction, which increases the chance of dysphagia. A decline in per os feeding and failure to flourish are expected. Regular oral intake is practiced in children aged 2 to 5 [11]. The possibility of respiratory problems following associated surgery must be considered. Feeding problems in these patients are exacerbated by structural anomalies such as a small jaw [12].

Although the exact nature of swallowing disorders in patients with *sporadic inclusion body myositis (s-IBM)* is unknown, we can identify some key clinical signs, such as prolapse of the pharyngeal muscles (PP) in the hypopharynx and upper esophageal sphincter (UES), as well as incomplete opening, leading to cricopharyngeal dysfunction [13]. Patients are frequently unable to detect dysphagia symptoms because the relevant processes occur subclinically. A video-fluoroscopy examination is helpful for diagnosis. The balloon dilatation procedure is used to supplement standard treatments. A 12-Fr balloon catheter is placed orally during fluoroscopy, and the balloon is then inflated within the UES. Alternatively, immunosuppressant or Botox infusion strategies have been demonstrated to be more effective than behavioral respiratory management techniques that do not alleviate dysphagia [13,14]. Consequently, pneumonia caused by aspiration or dehydration frequently results in death [15].

Dysphagia is the most common clinical manifestation of congenital *esophageal stenosis*, with the swallowing mechanism being the most affected by the esophageal stenosis. Esophagography and histological testing are required for diagnosis. Surgical treatment is a one-way street, regardless of the severity, location, or type of stenosis [16]. Dysphagia can be relieved by balloon dilatations or the insertion of a biodegradable polydioxanone stent (ELLA). More active stents include polypropylene silicate (PolyflexTM) [17]. Congenital esophageal stenosis is also common in the pediatric population, where children with feeding issues are at danger of being overlooked [18].

*Fahr illness* is characterized by oromandibular abnormalities and issues in muscular coordination that impair speech production. There is oropharyngeal dysphagia, which causes difficulties swallowing, and cervical dystonia, which has a significant impact on solid food consumption [19]. Deep brain stimulation (DBS) with bilateral implantation of two permanent electrodes in the hypothalamus nucleus (STN) is indicated therapeutically for the introduction of solid foods [20].

Swallowing and feeding problems occur with *Ataxia Telangiectasia* [21]. The gradual deterioration of neurological processes raises the risk of aspiration and pulmonary dysfunction [22]. Although research on oromotor and swallowing difficulties in these patients is scarce, we have important data, primarily from the valuable diagnostic test of video fluoroscopy (VFS). There is a shift in postural control during feeding, food retention in the oral cavity, slower oral transit time, and multiple swallowing. Some of the most important clinical signs reported are reduced muscle strength and tongue mobility, poor bolus ejection with difficulty regulating small amounts of water intraorally, cervical position change after swallowing, and respiratory distress (after ingestion of liquid puree), as well as laryngeal penetration for the liquid [23]. Specifically, the disorder occurs from early childhood in men, with the second decade of life being considered more prone to dysphagia issues [24]. Coughing and choking during meals are equally important clinical signs of dysphagia here. Therapeutically, percutaneous gastrostomy tube placement is recommended [25], but these patients do not exhibit safe expiratory patterns of pericoracic airflow [26]. Ataxia, hyperactivity, and ocular dysfunction impede the coordination of oral-motor and swallowing motions. In the long term, patients are pushed to high levels of malnutrition and, as a result, delayed development. Regular speech therapy sessions and tailored guidance on feeding routines and posture adjustments are essential. A gastrostomy can alleviate worries about food ingestion or poor food intake [24].

*Pontocerebellar Hypoplasia* is characterized by the presence of gastresophageal reflux disease (GERD), failure to thrive, and difficulty swallowing [27]. In the context of infants, the discussion pertains to difficulties in achieving an effective latch at the breast or bottle, resulting in reduced suction efficiency and suboptimal oral motor development. Regarding infants, there is talk of the inability to form a good seal at the nipple and consequent weak suction and poor oral motor function. The therapeutic options are determined by clinicians based on the nature and severity of the condition and any comorbidities [28].

Macroglossia and severe dysphagia are two of the most common clinical indications of *Pompe disease* [29]. In the pediatric community, patients with infantile-onset Pompe disease (IOPD) have mild to severe oropharyngeal dysphagia, poor oral stage skills, delayed swallowing onset, and persistent post-swallowing residue in the pharynx. Of course, the risk of aspiration pneumonia and chest infection should not be overlooked. Video-fluoroscopy is regarded as an appropriate diagnostic procedure. To improve school performance, patients are assessed every 6 months and receive concurrent speech therapy and dysphagia intervention sessions. A customized diet with non-oral feeding for up to 6 months is recommended as a therapy option, driving the decision to use a gastrostomy. ERT (Enzyme Replacement Therapy) is indicated for the late transition to oral feeding. Experimentation with variations in per os intake may be required over time [30]. Dysphagia in individuals with Biotin-Thiamine-Responsive Basal Ganglia Disease should be addressed promptly to mitigate the risk of progression to a comatose state.

It is characterized by poor eating, vomiting, and severe lactic acidosis [31]. Treatment with large doses of biotin and thiamine resolves symptoms within days [32].

In *Nemaline Myopathy*, there is a severe degree of dysphagia, notably in the pediatric population up to the third year of life, with a declining tendency in intensity with time. In at least 50% of cases, the gastrostomy technique is employed therapeutically, leading to improved facilitation of oral administration and enhanced nutritional management [33]. Botulinum toxin type A is a cost-effective and clinically efficient treatment option for cricopharyngeal dyskinesia. Oral massage (to strengthen the tongue and lips) is followed by passive sensory stimulation when the patient rejects oral stimuli and has trouble chewing reactions when food is offered [34].

These are part of the overall dysphagia treatment, which is provided twice a week in 30-min sessions and begins as soon as the VFSS reveals a decrease in aspiration. [33,35,36].

Food intolerance, hypersalivation, aspiration of oral secretions (attributable to bulbar dysfunction), gastresophageal reflux, and impaired oral occlusion are all anticipated clinical manifestations.

Food intolerance, increased saliva secretion, aspiration of oral secretions, gastresophageal reflux, and difficulty achieving complete oral closure are all predicted clinical symptoms. The Nissen tholoplasty procedure is preferred in instances of GERD with recurrent pneumonia. Shaker exercises, alongside buccinator and masseter muscle strengthening protocols, facilitate enhanced stability of the cervical, mandibular, and oromotor musculature, thereby contributing to the optimization of swallowing function.

Infants with impaired sucking reflexes are managed by the insertion of a nasogastric tube for nutritional support during the first year, followed by a gradual transition to pureed feeds.

Incomplete lip closure and an inability to control the tongue and jaw allow the bolus to remain in the oral cavity as a residue. There is a large delay in elicitation in the pharyngeal stage, with no laryngeal elevation or epiglottic movement and no cough response. Furthermore, the masseter and pterygoid muscles atrophy, while insufficient laryngeal vestibule closure leads to penetration and aspiration. Because of the mild contraction, residue is also observed in the pharyngeal area. Infants frequently refuse to breastfeed. They exhibit comparable behavior when eating solid food with a spoon (e.g., oatmeal). The significance of invasive treatments for this population has been established (albeit the post-surgical course cannot be predicted) [37].

*Esophageal atresia* is a disease with well-studied esophageal dysphagia. It is caused by structural anomalies (strictures, dyskinesia, and mucosal inflammation). Feeding problems are reported by a large percentage of esophageal atresia patients (up to 85%). Clinical indications such as oropharyngeal dysfunction, choking, vomiting, coughing, delayed feeding, pain and regurgitation, fear, and avoidance of solid foods are most seen in youngsters [38]. In the case of adolescents, complaints about food intake are recorded, and their energy intake is considered below established norms [39]. Any intolerance to feeding through a gastrostomy tube could be the cause.

To manage these, the clinician focuses on reducing aspiration, reducing full-column reflux in the oropharynx, reducing esophageal posture, dilating stenoses, and maintaining—with patience—oral feeding for continued practice of relevant skills with appropriate modifications [38]. Long-term gastresophageal reflux disease (GER), respiratory discomfort, and tracheomalacia are caused by esophageal dysphagia. Postoperative dysphagia is considered more problematic because the causes have not been precisely characterized, and anastomotic strictures have been implicated [40]. Upper GI and manometry (to analyze the physiology and architecture of the esophageal body and esophagogastric junction (EGJ) are indicated for diagnostic purposes [39,41,42].

The videofluoroscopic swallowing study (VFSS, to evaluate the causes of oropharyngeal dysphagia), the upper gastrointestinal contrast study (for esophageal strictures), and the esophageal motility test (to detect peristaltic esophageal movement) are all significant diagnostic procedures. These are supplemented by an esophagogram, a pH impedance study, and an esophagoscopy with biopsy (per the ESPGHAN-NASPGHAN criteria).

Adults with *oropharyngeal dysphagia* exhibit cyanotic episodes before, during, or after meals, as well as arching of the back, wet eyes, a burning sensation in the chest, or noisy breathing (wheezing). Laryngeal fissures, paralysis of the voice cords, changes in neuromuscular control, and departures from developmental patterns in the swallowing process distinguish this dysphagia from its equivalents (other illnesses). Transpyloric feeding can help to lessen the amount of reflux. The option of tholoplasty (to minimize refusal-aversion to feeding) was rejected since it was thought that the patient would be more likely to develop dysphagia postoperatively. Finally, changing the feeding schedule, using cyproheptadine (which stimulates the appetite), and eating dense foods can help alleviate symptoms [43].

The symptoms of *Wallenberg syndrome* include unilateral paralysis of the soft palate, larynx, and throat, as well as dysphagia and hoarseness. The following symptoms have been reported: a feeling of a foreign body after eating, difficulties swallowing saliva and mixed consistencies of food with a sudden feeling of choking, increased sputum, and cough induction (with ineffective clearance). Polyphagia, polydipsia, and unexplained weight loss with instability in the lower limbs are observed after the onset of dysphagia, accompanied by mucous membrane dryness, a lack of gag response, and unprovoked hiccups. Diagnostic tests include a CT scan of the neck’s soft tissues (to look for a foreign body or external compression), a complete neurological evaluation (to rule out the potential of infection), and a bedside swallowing test.

The orofacial examination reveals morbidities such as unilateral glottic and tongue deviation and vocal cord paralysis [44]. Therapeutically, a transient percutaneous endoscopic gastrostomy is used, whereas Romberg/HINTS tests help to relieve symptoms [45]. Biofeedback technique in functional electrical stimulation (FES) is the most sophisticated treatment for dysphagia within the syndrome. This technique uses visual feedback to guide the patient through the basic swallowing movement patterns.

The biofeedback technique optimizes the swallowing function (in time and intensity) after learning the basic sensorimotor processes by using surface electromyography (EMG) of the supraspinal muscles to strengthen and elevate the vitreo-laryngeal complex. The procedures described above are integrated utilizing specialized equipment. A training device with two subsystems is shown: the RehaIngest, which analyzes certain areas of swallowing (laryngeal elevation, speed, and duration) and activates the action of the second subsystem, and the RehaMove (which strengthens the supraspinatus muscles). The patient is taught the proper swallowing posture while remaining aligned. When paired with other techniques (Mendelsohn maneuver), biofeedback games and electrical stimulation aid in preserving the airways [46].

A small amount of juice in soaked gauze and boluses administered with a small spoon are used to initiate swallowing, and stimulation electrodes are often placed under the jaw [46]. In general, dysphagia is present in 100% of individuals with this syndrome and resolves between 4–10 weeks with appropriate treatment, such as facial oral tube therapy (F.O.T.T.) [44,47,48].

In conditions where resistant reflux with dysphagia occurs, the likelihood of *IgG4 sclerosing disease* of the esophagus should be addressed for suitable intervention selection [49], such as mycophenolate mofetil (to reduce continuous esophageal dilations). After meals, abdominal pain and nausea become worse [50,51]. Glucocorticoids treat the abovementioned conditions (mycophenolate mofetil, for example, to reduce continuous esophageal dilations) [49]. After meals, abdominal pain and nausea become worse [50,51]. Prednisolone is used daily to treat swallowing difficulties [52]. The patient repeatedly feels a food bolus, which lasts until corticosteroids and methotrexate are administered. A nasogastric tube is advised for general dysphagia control [50,52]. Esophageal involvement is rare, with fewer than 20 cases documented in the literature. The recommended diagnostic approach includes endoscopic evaluation, which can reveal distinct findings such as significant stenosis, nodular formations, and fibrotic alterations at the gastresophageal junction [51,53].

The condition must be distinguished from infectious esophagitis [52]. The most common clinical manifestations are malignant lesions in the oral cavity, which cause increasingly severe dysphagia. The patient’s symptoms are alleviated by taking diclofenac thrice daily [50,53]. Enalaplasty, esophagectomy-esophagogastrectomy, or ESD are the choice procedures [51].

*Progressive dysphagia* (often accompanied by achalasia) is related to a parasympathetic nervous system abnormality in Sjögren’s illness. The clinical manifestations encompass the pharynx, esophagus, and gastroesophageal reflux [54]. Xerostomia, foreign body sensation in the throat after a meal (resulting in sputum of under-eaten food), trouble swallowing solids, choking on liquids, stomach acid reflux, delayed swallowing reflex, or choking are all described symptoms. Dysphagia is classified as oropharyngeal (due to salivary gland dysfunction or mucositis) or esophageal (with decreased esophageal motility due to esophageal myenteric plexus loss), with a 60% prevalence of self-reported dysfunction and coexisting duodenitis is possible. Finally, the typical peristaltic movement of the esophageal body during swallowing is absent and is observed pooling in the pyriform sinuses. Early steroid treatment and correct swallowing training approaches for regaining peristaltic esophageal movement lead to dysphagia alleviation [55].

*Sandifer syndrome* is an uncommon consequence of gastresophageal reflux disease and diaphragmatic hernia (1% in children). Muscular contractions in the throat area after a meal, upper gastrointestinal (GI) tract problems, rumination, dystonic posture, and inability to feed (mostly in newborns) are recorded as clinical indications of dysphagia. The absence of rhythmic clonic spasms distinguishes the illness from epilepsy, and it is misdiagnosed with neuromuscular disorders. Laboratory studies are not regarded as efficient diagnostically. To remove acid and relieve discomfort, training to maintain an upright position for a few minutes after feeding or a position with the head tilted laterally (to lessen pressure on the esophagus) is recommended.

Alternatively, receptor antagonists and PPIs such as histamine, ranitidine, and cimetidine (with the main disadvantage of producing tachyphylaxis) are considered the best pharmacological alternatives. Tholoplasty surgery (enlargement of the intra-abdominal section of the esophagus) is indicated for long-term relief. Simultaneously, advising parents to reduce the number and frequency of meals while minimizing secondhand smoke exposure can be significant. Furthermore, reflux can be characterized as primary (due to lower esophageal sphincter dysfunction) or secondary (due to upper gastrointestinal tract dysfunction) [56].

*Foix-Chavany-Marie Syndrome (FCMS)* is related to progressive multifocal leukoencephalopathy (PML) and affects involuntary control of the face muscles, oromandibular mechanism, pharynx, and larynx. With extended PML and a lack of cricopharyngeal muscle dilatation, there is dysfunction of the oral and pharyngeal stages of swallowing and prolonged apnea while swallowing, followed by aberrant expiration (abnormal breath-swallow coordination).

An electrokinetic/electromyographic swallowing study (EES) or endoscopic examination of swallowing with fiber optics (FEES) is deemed diagnostically helpful. There is also an inability to open the mouth and protrusion of the tongue, retention of the bolus within the oral cavity and tongue-assisted advancement to the palate (regardless of bolus composition: water or jelly), a weak cough reflex, passive dropping (“posterior leakage”) of the bolus into the pharynx, and weakened supraspinal muscle activity [57].

There is also an inability to open the mouth and protrusion of the tongue, retention of the pylorus within the oral cavity and tongue-assisted advancement to the palate (regardless of pylorus composition: water or jelly), a weak cough reflex, passive dropping (“posterior leakage”) of the pylorus into the pharynx, and weakened supraspinal muscle activity.

*Aortic dysphagia* (often associated with the impending development of an aorto-esophageal fistula) is an uncommon disorder caused by a thoracic aortic anomaly and subsequent external compression in the esophagus. A massive para-aortic hematoma compresses the esophagus. The patient has dysphagia on solid foods that started suddenly and has progressively worsened. In the absence of cardiorespiratory distress, the dysphagia rapidly advances to include difficulty with liquids, accompanied by apparent chest pain.

The most relevant diagnostic test, esophagogastroduodenoscopy, reveals esophageal dilatation and remaining food. An abnormal vascular stricture extrinsically compresses the esophagus, resulting in a unique type of dysphagia, particularly in hypertensive women with small stature, advanced age, and kyphosis [58].

*D’Allgrove syndrome/triple A syndrome* is an autosomal recessive genetic disease with the main clinical indications being pigmented slate on the dorsum of the tongue, palate, gums, and inner surface of the cheeks, as well as soft palate shrinkage with undesired tongue spasms [59]. Furthermore, the location of the esophagus is like that of achalasia. Manometry of the esophagus, a barium swallow test, and a nutritional assessment are all advised as diagnostic procedures. Therapeutic options include esophageal dilatation, dietary changes, or surgery [60]. Alternatively, balloon dilatation of the esophago-gastric junction is thought to be more cost-effective than surgery in the treatment of dysphagia or undesired nocturnal cough. Nifedipine is advised for the treatment of achalasia [61,62].

In general, EBD is considered the traditional technique of illness management, while POEM has recently superseded it [59]. The syndrome has been observed in the pediatric population, together with dysphagia and xerostomia due to saliva production malfunction, severe caries and tooth loss, nasal speech, and hypoglycemic crises [63]. Hydrocortisone and artificial tear saliva are recommended to treat the aforementioned conditions [64].

Diffuse idiopathic skeletal hyperostosis (DISH) in *Forestier syndrome* is a rheumatological disease characterized by osteophytes’ growth in the neck and expressed by otolaryngological symptoms, most notably difficulty swallowing and breathing. The presence of an osteophyte at the C2–3 level causes dysphagic episodes. The latter are removed via excision of the osteophytes. The disease has a male predisposition, with an age incidence above 65. The patient frequently experiences trachealgia and wheezing, and dysphagia affects 0.6–1.0% of cases owing to esophageal constriction in the lower portions of the neck.

As a result, esophageal blockage and laryngeal nerve injury are the most common causes of dysphagia. A barium swallow test can assist in determining the degree of esophageal compression. Traditional treatment techniques such as nonsteroidal anti-inflammatory or muscle relaxants, dietary modifications, and anti-reflux therapeutic techniques are used. Surgical intervention is preferred in people who are resistant to the preceding treatments [65].

*Joubert syndrome* is diagnosed at 33 months of age in children and is distinguished by gradually deteriorating dysphagia (beginning with solids) that is asymptomatic in the first few months. Subsequently, there is a feeling of a foreign body after meals, reduced oral intake, and lack of a gag response. Typically, the oral cavity is unharmed [66].

*SAPHO* is a syndrome of hymenitis, acne, pustulosis, hyperostosis, and osteoarthritis syndrome. Fluids collect in the soft tissues of the neck and chest, while the sternum bones deteriorate catastrophically. CT and MRI of the neck, as well as retropharyngeal fluid accumulation and anterior collection obstructing the trachea, are deemed suitable studies [67].

Orofacial symptoms include osteomyelitis of the mandible, which necessitates mandibular surgery. Intense, recurring pain is centered in the mouth cavity, along with hypesthesia and structural deformation. Severe pharyngeal pain, hypophonia (owing to vocal cord paralysis), saliva stagnation in the posterior region, and arytenoid tremor have been observed on rare occasions. Oral hygiene is used to assist in preventing bacteria from entering the lungs in cases of aspiration; however, it should be implemented as a prophylactic measure.

As a preventive step, oral hygiene and tooth extraction are indicated as therapeutic. Methotrexate at a modest dose alleviates dysphagia and cutaneous symptoms [68,69].

*Collet-Sicard Syndrome (CSS)* is an uncommon disease characterized by unilateral paralysis of cranial nerves IX, X, XI, and XII. It happens after an injury to the jugular foramen (controls cranial nerves connected with the normal cough response) or the sublingual canal (controls the nerve involved in tongue motions). The chief clinical indications of dysphagia are rightward deviation of the tongue, leftward deviation of the pharynx, and a fixed palate. A fixed or immobile palate indicates significant structural impairment or dysfunction of the palatal musculature, typically associated with the involvement of cranial nerves IX (glossopharyngeal) and X (vagus), which provide motor innervation to these muscles. Failure of the palate to elevate during swallowing or phonation compromises nasopharyngeal closure, increasing the risk of dysphagia and aspiration [70].

The patient is unable to swallow his saliva. The primary therapeutic goals are the resolution of dysphagia and the normalization of the tongue in the midline. For 3–6 months, nasogastric tube feeding and percutaneous endoscopic gastrostomy (PEG) tube implantation are clinically advised [70].

*Wolfram Syndrome* is an extremely rare hereditary condition that causes excessive blood sugar levels. Dysphagia and loss of taste are important clinical indications that, along with balance problems, indicate brainstem injury. The condition progresses in a staggered pattern up to the fourth decade of the patient’s life when severe dysphagia causes premature death [71]. The major goal of treatment is to stabilize the intracellular calcium concentration, and cricopharyngeal myotomy is advised for dysphagic episodes [72,73].

*Rubinstein-Taybi syndrome* is a rare (1 in 100,000 infants) polymorphic autosomal dominant genetic disease with equal racial distribution. Its predominant symptoms are gastrointestinal in nature, with difficulty feeding and gastresophageal reflux disease (GERD). Second, if not treated immediately, the risk of problems such as failure to thrive and creating massive (up to 32 cm) strictures through esophageal tissue increases. It is worth mentioning that dysphagia in this context primarily refers to solid foods, but esophageal issues might cause recurring respiratory problems and metacryptic plexuses. Esophagogastroduodenoscopy (EGD) is the recommended diagnostic test, while endoscopic balloon dilatation is the primary treatment approach for esophageal strictures [74].

Oropharyngeal dysphagia [75] occurs in the setting of *Potocki-Lupski syndrome* (a rare chromosomal anomaly caused by partial reduplication of chromosome 17 [76], which in the long run causes developmental issues (mainly gross motor delays). Significantly reduced weight and height, micrognathia that can lead to or be associated with poor occlusion, dysphagia characterized by lack of coordination between swallowing stages, and gastresophageal reflux disease (GERD) are frequently observed in the pediatric population. Clinical manifestations also include failure to thrive, lingual weakness, and the presence of a high-arched palate [76,77].

In this case, tracheal intubation with a feeding tube is indicated in the short term [78]. A video laryngoscope examination and neuromuscular blockade monitoring are helpful diagnostic tools. Preventing pulmonary aspiration is advised both perioperatively and postoperatively. Supraglottic airways could be beneficial [77].

Another uncommon (0.1–0.28%) [79] medical condition is the black esophagus, also known as *acute esophageal necrosis syndrome (AEN)*, which is caused by diffuse or encrusted black staining of the esophageal mucosa (due to ischemia necrosis). Candidates are elderly persons with comorbidities, and mortality rates are exceedingly high [80]. Diagnostic tools include endoscopic evaluations, histological esophageal biopsies, and esophagogastroduodenoscopy. The condition presents acute epigastric pain [79], hematemesis [81], and complications such as stenoses, perforations, mediastinitis, and gastrointestinal obstruction causing malnutrition [80,82]. Management involves acid suppression, airway protection via intubation, and vasoconstrictor use. Solid fasting is preferred over fluids to minimize transfusion needs. Nasogastric tubes are avoided due to perforation risk, and severe esophageal strictures may require transesophageal surgery or bougie dilation [79]. SLPs are critical in documenting clinical symptoms and performing objective instrumental assessments.

*Kaposi’s Sarcoma (KS)* is a vascular tumor caused by infection with human herpesvirus 8. The oral mucosa is typical; however, the tonsils are infrequently affected (they become hypertrophied and develop a distinctive purple coloring). Common complications include dysphagia, uncomfortable eating, and weight loss. Substantial expansion of the Waldeyer’s ring is required to increase the likelihood of enlarged cervical lymph nodes. Including sirolimus in the treatment regimen helps normalize the oropharyngeal lesions. According to research, cricothyrotomy is superior to “traditional” tracheostomy [83,84]. Laryngeal KS causes airway blockage. Direct laryngoscopy, biopsy, and histological testing are used to diagnose it. A nodular lesion develops from the pharyngeal walls and extends to the true vocal cords [84].

*Tapia syndrome* develops due to laryngeal and sublingual nerve injury during postoperative general anesthesia. Hoarseness in conjunction with eating difficulties, difficulty swallowing, and vocal cord paralysis. It is explored with flexible laryngoscopy, modified barium swallowing, and a neck CT scan. Feeding difficulties are characterized by mild to moderate pharyngeal dysphagia and aspiration of thin and viscous fluids. Injection of carboxymethyl cellulose implant into the vocal cords becomes beneficial therapeutically. Steroids and speech therapy sessions are also suggested as alternatives. Cricothyroid catheter, dexamethasone, dipyridamole, electrical stimulation therapy, NGT, and esophageal balloon dilatation are used for more dramatic effects. The syndrome is classified into three classes based on the severity of the symptoms, with the third being connected with difficulty swallowing liquids and solids. Clinical signs include ecchymosis/weakness in the right soft palate and right side of the tongue, hemiplegia/tongue swelling and deviation to the left, neck and throat pain, submucous hemorrhage of the left vocal cord, difficulty swallowing, laryngeal spasm, drip deformity, dry throat, unilateral swelling of the tongue, choking, inadequate closure of the glottis, coughing, and tongue bite wounds; the oral cavity is hypersensitive, and there is no gag reaction. Clinical signs include ecchymosis/weakness in the right soft palate and right side of the tongue, hemiplegia/tongue swelling and deviation to the left, neck and throat pain, submucous hemorrhage of the left vocal cord, difficulty swallowing, laryngeal spasm, drip deformity, dry throat, unilateral swelling of the tongue, choking, inadequate closure of the glottis, coughing, and tongue bite wounds [85].

Finally, *König’s syndrome* is characterized by abdominal pain after meals, constipation, flatulence, and weight loss. Dysphagia and indigestion are regarded as expected outcomes. It is frequently caused as a long-term postoperative complication after Roux-en-Y gastric bypass surgery. The barium swallow test, or esophago-gastroduodenoscopy (OGD), is preferred to investigate dysphagia. Any surgical procedure is thought to be more effective therapeutically [86]. Next, we provide a summary table presenting various diseases and their key characteristics in a concise, aggregated format. This approach provides a clear and structured overview of diseases and their defining features (Table 2).

## 4. Discussion

The current systematic review aims to investigate the complex interface between RDS and dysphagia, with a primary objective of elucidating pathophysiological mechanisms, identifying rare conditions and syndromes that carry the highest risk for feeding and swallowing disorders, characterizing typical clinical presentations, and outlining assessment and treatment strategies that leverage the contributions of a multidisciplinary team [87]. We have arrived at several noteworthy points in addressing the specific research questions posed in our study. Next is a detailed discussion of each question from this study, including future research priorities in dysphagia rehabilitation and support, limitations, ethical considerations, and scientific gaps that require further research.

### 4.1. Core Pathophysiological Processes Driving Dysphagia in RDS

In RDS, dysphagia often arises from a convergence of neuromuscular, structural, and sensory abnormalities. Degeneration of neural pathways or muscles disrupts the precise coordination needed for swallowing. Sensory deficits further impair protective reflexes. Together, these factors compromise the smooth transfer of food and liquids, leading to difficulty swallowing.

The presence and severity of swallowing problems vary among disease subtypes and disease stages. The interaction of these factors may be further complicated by systemic disease processes, nutritional deficiencies, and compromised respiratory function, leading to a complex clinical picture that can evolve over time and adversely impact quality of life. Specifically, the findings highlight the multifactorial nature of dysphagia in RDS, underscoring the need for differentiated, individualized management approaches.

Although the literature is composed of heterogeneous samples and, in some instances, isolated case reports, cumulative evidence suggests that dysphagia is not merely a coincidental comorbidity but likely a common and frequently underrecognized clinical manifestation in many RDS associated with structural and functional deficits of the swallowing mechanism.

### 4.2. RDS Commonly Associated with Dysphagia and Their Effects on Swallowing

Several RDS are often linked to dysphagia and its impact on swallowing. Rare neuromuscular disorders, progressive muscular dystrophies, connective tissue disorders, neurodegenerative conditions, and congenital craniofacial malformations appear in the literature as associated with feeding and swallowing difficulties.

### 4.3. Clinical Manifestations of Dysphagia in Rare Diseases: Common Symptoms and Presentations

Dysphagia in patients with RDS typically presents as difficulty in bolus formation, prolonged oral transit, nasopharyngeal regurgitation, and episodes of coughing or choking during or after swallowing. Additionally, dysphagia commonly manifests as reduced lingual strength resulting from impaired muscle strength or coordination, altered sensation, decreased airway protection, structural anomalies, delayed swallow initiation, reduced bolus transit, and an increased risk of aspiration.

### 4.4. Clinical Patterns and Key Symptoms of Dysphagia in RDS

Some patients report changes in tolerance to food consistency, unintentional weight loss, and a persistent sensation of food “sticking” in the throat. Clinically, abnormal swallow timing, valleculae or pyriform sinuses residue, and aspiration events observed through instrumental evaluation are common findings. These symptoms are often subtle in the early stages of the disease and may be overshadowed by other systemic manifestations, thus delaying diagnosis by the multidisciplinary team [88].

### 4.5. Evaluating Dysphagia in RDS: Key Diagnostic Methods and Specialized Tools

Although comprehensive clinical swallowing evaluations remain the initial step, instrumental assessments, including the videofluoroscopic swallowing study (VFSS) and the fiberoptic endoscopic evaluation of swallowing (FEES), represent gold-standard methods for characterizing dysphagia in RDS. Specialized speech-language pathologists conduct these procedures in the presence of a radiologist.

High-resolution manometry provides nuanced information regarding pressure dynamics throughout the swallowing apparatus, facilitating targeted interventions. [89]. Emerging imaging technologies, such as dynamic magnetic resonance imaging and three-dimensional reconstructions, alongside advanced electrophysiological techniques, hold promise for detecting subtle biomechanical deficits. The complexity of dysphagia associated with RDS underscores the importance of a multidisciplinary diagnostic approach, incorporating expertise from otolaryngology, neurology, gastroenterology, and speech-language pathology [90].

### 4.6. Innovative, Personalized Dysphagia Therapies for Rare Diseases: Success and Advances

Today, innovative, targeted therapies are emerging for the management of dysphagia in populations with RDS. Treatment strategies range from traditional compensatory techniques, such as postural adjustments and dietary modifications, to more intensive rehabilitative approaches, including specialized exercise regimens, neuromuscular electrical stimulation (NMES), and biofeedback methods that enhance motor learning and neuromuscular control. Emerging data support the selective use of NMES and surface electromyography (sEMG) biofeedback as adjuncts to conventional therapy, with certain rare disease populations demonstrating notable improvements in swallowing function and safety. Personalized approaches that tailor interventions to disease progression, individual deficits, and patient-specific profiles are expected to yield optimal outcomes. Although clinical data remain limited, genetic therapies, regenerative medicine, and advanced neuromodulation techniques are promising future treatments. Moreover, tailoring interventions to genetic, phenotypic, and disease-specific factors is increasingly recognized as essential for optimizing outcomes, particularly with the involvement of specialized speech-language pathologists [90].

### 4.7. Dysphagia in RDS: Challenges and Research Needs

The current literature on dysphagia in RDS is fragmented, lacking well-designed longitudinal studies and relying heavily on case reports. Variability in disease trajectories, phenotypic expression, and access to suitable diagnostic tools hinders the establishment of standardized guidelines [91]. Limited research on prognostic biomarkers and the long-term effectiveness of innovative therapies constrains evidence-based practice. Greater emphasis on large-scale, multicenter collaborations and data sharing is crucial to bridging these gaps, as is the development of standardized outcome measures tailored to the rare disease population [91].

### 4.8. Future Research Priorities in Dysphagia for RDS

Future priorities should include developing robust, validated assessment tools sensitive to subtle swallowing deficits. Incorporating patient-reported outcomes will enhance clinical relevance [92]. Research into underlying mechanisms that guide targeted interventions and the integration of precision medicine approaches that consider genetic and molecular signatures could optimize therapeutic results. [93]. Interdisciplinary collaborations, improved awareness, and enhanced clinician training will be critical to ensure that patients with RDS receive timely, evidence-based management of their dysphagia [94]. Key research objectives encompass the establishment of standardized diagnostic methodologies, the identification of disease-specific biomarkers for dysphagia, the execution of large-scale clinical trials to evaluate emerging treatment modalities, and the cultivation of interdisciplinary collaborations aimed at developing personalized, evidence-based therapeutic protocols [95].

In conclusion, this review emphasizes that dysphagia represents a clinically significant yet insufficiently characterized complication across numerous RDS [5]. Although emerging diagnostic and therapeutic modalities demonstrate potential, ongoing rigorous investigations remain necessary to refine these interventions, develop robust evidence-based guidelines, strengthen multidisciplinary collaborations—particularly with highly trained speech-language pathologists—and ultimately improve the health-related quality of life for affected individuals.

### 4.9. Limitations

Furthermore, dysphagia presents differently across these rare diseases, with substantial variation in severity and the types of deficits associated with structural versus neurological lesions. This heterogeneity complicates the development of standardized therapeutic approaches and limits the ability to draw broad conclusions regarding the management of dysphagia in these cases. Treatment interventions also differ depending on the clinical context, the expertise of the practitioner, and available resources. Moreover, the lack of longitudinal studies hinders the evaluation of the long-term efficacy of revised therapies, while limited accessibility to these treatments impacts their overall applicability and effectiveness.

### 4.10. Ethical Considerations

Ethical research on RDS should incorporate patient and caregiver perspectives to better align study designs with the needs and values of the population. This participatory approach ensures that the research remains patient-centered and respectful of their experiences.

### 4.11. Scientific Gap—Future Research

The systematic review highlights critical scientific gaps and areas requiring further investigation to enhance the understanding and management of dysphagia in rare diseases. A key step is the detailed mapping of the pathophysiological mechanisms underlying dysphagia. Addressing these gaps is essential for improving diagnostic accuracy, therapeutic efficacy, and patient outcomes in these specialized populations. This goal necessitates validating reliable, syndrome-specific diagnostic tools and protocols, including non-invasive methods. Additionally, comparative studies to identify the most effective diagnostic techniques for specific conditions will significantly strengthen clinical practice.

### 4.12. Conclusions

Dysphagia is a significant challenge in the management of rare diseases and syndromes, impacting morbidity, nutrition, and quality of life. This systematic review highlights the need for a multidisciplinary approach integrating advanced diagnostics, personalized therapies, and emerging technologies. Promising interventions, including tailored rehabilitation, nutritional optimization, pharmacological treatments, and minimally invasive surgeries, show potential to improve outcomes. However, gaps in understanding dysphagia’s pathophysiology in rare conditions necessitate focused research, standardized frameworks, and evidence-based protocols. By fostering innovation, interdisciplinary collaboration, and patient-centered care, we can address these challenges, advancing clinical practice and enhancing the lives of affected individuals.

## Figures and Tables

**Table 1:** Summary of Search Strategy and Inclusion Criteria According to PRISMA 2020.

Category	Details
Publication Type	Original articles, reviews, case studies
Language	English
Publication Date	Primarily within the last 10 years
Content	focusing on RDS and therapeutic approaches for dysphagia
Databases Searched	Science Direct, PubMed
Search Terms	“deglutition” OR “swallowing disorders” OR “dysphagia” OR “deglutition disorders” OR “swallowing difficulties” OR “rare disorders” OR “rare syndromes” OR “rare diseases” OR “case studies” AND (speech and language therapy OR pathology) AND “dysphagia management”
Case Studies	Global coverage, no geographical, racial, or age constraints
Research Gaps and Future Directions	Studies discussing current challenges, knowledge gaps, and research priorities in managing dysphagia in rare syndromes.

**Table 2:** Summary of Syndromes, Characteristics, and Clinical Management.

Reference	Type of Study (Research Article, Case Study, Review)	Syndrome/Disease/Condition	Gene	Incidence	Etiology (Genetic, Neurogenic, Acquired)	Key Features	Dysphasia (Primary, Secondary)	Treatment/Management	Speech Language Therapy
[7]	Research Article	Plummer-Vinson Syndrome (PVS)	-	Rare	Acquired	Dysphagia, GERD, anemia, weight loss	Primary	Iron replacement, esophageal dilation	Not explicitly mentioned
[8,9]	Research Article	Niemann-Pick Disease Type C (NP-C)	NPC1, NPC2	Rare	Genetic	Lipid accumulation, delayed swallowing reflex	Primary	Miglustat, aspiration risk management	Focus on swallowing stabilization.
[10]	Research Article	Stüve-Wiedemann Syndrome	-	Rare	Genetic	Dysphagia, cervical root dysfunction	Primary	Aspiration management	Tailored feeding protocols
[11,12]	Case Study	Moebius Syndrome	-	Rare	Genetic/Neurogenic	Cranial nerve paralysis, jaw anomalies	Primary	Surgical and structural management	Feeding protocols for children
[13,14,15]	Research Article	Sporadic Inclusion Body Myositis (s-IBM)	-	Rare	Acquired	Pharyngeal muscle prolapse, UES dysfunction	Secondary	Balloon dilation, immunosuppressants	Speech therapy with VFSS guidance
[16,17,18]	Review Article	Congenital Esophageal Stenosis	-	Rare	Genetic	Esophageal stenosis, feeding issues	Primary	Surgical intervention	Feeding assistance for children
[19,20]	Research Article	Fahr Syndrome	-	Rare	Genetic	Oromandibular issues, dysphagia	Primary	DBS, solid food introduction	Oropharyngeal strengthening
[21,22,23,24]	Review Article	Ataxia Telangiectasia	ATM	Rare	Genetic/Neurogenic	Dysphagia, aspiration risk, poor bolus control	Primary	Posture adjustment, gastrostomy	Regular therapy and bolus training
[27,28]	Research Article	Pontocerebellar Hypoplasia	-	Rare	Genetic	GERD, poor suction in infants	Primary	Feeding intervention	Development of oral-motor function
[29,30]	Research Article	Pompe Disease (Infantile Onset)	GAA	Rare	Genetic	Macroglossia, severe dysphagia	Primary	ERT, gastrostomy, diet modification	Speech therapy every 6 months
[31,32]	Review Article	Biotin-Thiamine-Responsive Basal Ganglia Disease	SLC19A3	Rare	Genetic	Poor eating, lactic acidosis	Primary	Biotin and thiamine supplements	Focus on swallowing safety
[33,34,35,36]	Research Article	Nemaline Myopathy	-	Rare	Genetic	Severe dysphagia, oral-stage difficulties	Primary	Botulinum toxin, gastrostomy	Oral massage, sensory stimulation
[38,39,40]	Review Article	Esophageal Atresia	-	Rare	Genetic	Dysphagia, GERD, regurgitation	Primary	Surgical gastrostomy	Feeding skill maintenance
[44,45,46,47]	Case Study	Wallenberg Syndrome	-	Rare	Neurogenic	Dysphagia, soft palate paralysis	Primary	Biofeedback, gastrostomy	Biofeedback-based swallowing
[50,51,52,53]	Research Article	IgG4 Sclerosing Disease of the Esophagus	-	Very Rare	Acquired	Dysphagia, reflux, nausea	Secondary	Glucocorticoids, dilation	Feeding routine adjustments
[54,55]	Research Article	Sjögren’s Syndrome	-	Rare	Neurogenic	Xerostomia, GERD, delayed reflex	Secondary	Steroid treatment	Swallowing training techniques
[56]	Research Article	Sandifer Syndrome	-	Rare	Neurogenic/GERD	Dystonia, feeding refusal	Secondary	Upright posture, surgical, PPIs	Parent guidance on feeding
[57]	Research Article	Foix-Chavany-Marie Syndrome	-	Rare	Neurogenic	Oral bolus retention, dysphagia	Primary	Electromyography-guided therapy	Sensorimotor training
[58]	Research Article	Aortic Dysphagia	-	Very Rare	Acquired	Thoracic aortic anomaly, esophageal compression	Secondary	Endoscopy, esophageal dilation	Assessment and management of feeding techniques
[59,60,61,62,63,64]	Review Article	D’Allgrove Syndrome	AAAS	Rare	Genetic	Xerostomia, nocturnal cough, dysphagia	Primary	Esophageal dilation, artificial saliva, surgery	Focused therapy on oral-motor dysfunction
[65]	Research Article	Diffuse Idiopathic Skeletal Hyperostosis (DISH)	-	Rare	Acquired	Osteophyte growth, dysphagia	Secondary	NSAIDs, muscle relaxants, osteophyte excision	Dysphagia therapy with posture optimization
[66]	Case Study	Joubert Syndrome	-	Rare	Genetic	Dysphagia, poor gag reflex	Primary	Nutritional assessment, feeding techniques	Oral coordination therapy
[67,68,69]	Research Article	SAPHO Syndrome	-	Rare	Acquired	Retropharyngeal fluid, osteomyelitis	Secondary	Methotrexate, mandibular surgery	Prevention-focused therapy
[70]	Review Article	Collet-Sicard Syndrome	-	Rare	Neurogenic	CN IX-XII paralysis, fixed palate, dysphagia	Primary	PEG tube, nasogastric feeding	Swallowing technique enhancement
[71,72,73]	Review Article	Wolfram Syndrome	WFS1	Rare	Genetic	Dysphagia, brainstem injury, balance issues	Primary	Cricopharyngeal myotomy, calcium stabilization	Aspiration risk management
[74]	Case Study	Rubinstein-Taybi Syndrome	CREBBP, EP300	02:40, 0	Genetic	GERD, esophageal strictures	Secondary	Endoscopic balloon dilation	GERD management with feeding adjustments
[75,76,77,78]	Research Article	Potocki-Lupski Syndrome	Duplication 17p11.2	Rare	Genetic	GERD, dysphagia, developmental delays	Primary	Tracheal intubation, aspiration prevention	Oral-motor training
[79,80,81,82]	Research Article	Acute Esophageal Necrosis (AEN)	-	0.1–0.28%	Acquired	Black esophagus, epigastric pain, hematemesis	Primary	Acid suppression, vasoconstrictors, surgery	SLP-led objective symptom assessments
[83,84]	Case Study	Kaposi’s Sarcoma	HHV-8	Rare	Viral-induced	Tonsillar hypertrophy, dysphagia, weight loss	Secondary	Sirolimus, laryngoscopy, cricothyrotomy	Speech therapy for oropharyngeal lesions
[85]	Research Article	Tapia Syndrome	-	Rare	Neurogenic/Acquired	Vocal cord paralysis, mild-to-moderate dysphagia	Secondary	Steroids, electrical stimulation, feeding techniques	Strengthening of swallowing mechanism
[86]	Review Article	König’s Syndrome	-	Rare	Acquired (postoperative)	Abdominal pain, indigestion, dysphagia	Secondary	Surgical intervention	Feeding technique restoration

## Data Availability

Not applicable.
